# Recommendations for Assessment of Environmental Exposures in Longitudinal Life Course Studies Such as the National Children's Study

**DOI:** 10.3389/fped.2021.629487

**Published:** 2021-04-29

**Authors:** Susan Marie Viet, Michael Dellarco, Edith Chen, Thomas McDade, Elaine Faustman, Sean Brachvogel, Marissa Smith, Rosalind Wright

**Affiliations:** ^1^Westat, Rockville, MD, United States; ^2^Eunice Kennedy Shriver National Institute of Child Health and Human Development, Bethesda, MD, United States; ^3^Department of Pscychology, Northwestern University, Evanston, IL, United States; ^4^Department of Anthropology, Northwestern University, Evanston, IL, United States; ^5^University of Washington, Seattle, WA, United States; ^6^Icahn School of Medicine at Mount Sinai School, New York, NY, United States

**Keywords:** exposure assessment, longitudinal research, environmental exposures, pediatric longitudinal research, psychosocial environment

## Abstract

An important step toward understanding the relationship between the environment and child health and development is the comprehensive cataloging of external environmental factors that may modify health and development over the life course. Our understanding of the environmental influences on health is growing increasingly complex. Significant key questions exist as to what genes, environment, and life stage mean to defining normal variations and altered developmental trajectories throughout the life course and also across generations. With the rapid advances in genetic technology came large-scale genomic studies to search for the genetic etiology of complex diseases. While genome-wide association studies (GWAS) have revealed genetic factors and networks that advance our understanding to some extent, it is increasingly recognized that disease causation is largely non-genetic and reflects interactions between an individual's genetic susceptibility and his or her environment. Thus, the full promise of the human genome project to prevent or treat disease and promote good health arguably depends on a commitment to understanding the interactions between our environment and our genetic makeup and requires a design with prospective environmental data collection that considers critical windows of susceptibility that likely correspond to the expression of specific genes and gene pathways. Unlike the genome, which is static, relevant exposures as well as our response to exposures, change over time. This has fostered the complementary concept of the exposome ideally defined as the measure of all exposures of an individual over a lifetime and how those exposures relate to health. The exposome framework considers multiple external exposures (e.g., chemical, social) and behaviors that may modify exposures (e.g., diet), as well as consequences of environmental exposures indexed via biomarkers of physiological response or measures of behavioral response throughout the lifespan. The exposome concept can be applied in prospective developmental studies such as the National Children's Study (NCS) with the practical understanding that even a partial characterization will bring major advances to health. Lessons learned from the NCS provide an important opportunity to inform future studies that can leverage these evolving paradigms in elucidating the role of environment on health across the life course.

## Introduction

### Measuring the Environment

The etiology of health and well-being is increasingly recognized to result from the complex interplay of environmental influences operating at multiple levels, including the individual, the home and family, the neighborhood/community, and beyond (1, 2). As part of this growing complexity, evidence suggests that connections between health and economic well-being are embedded within the larger context of people's lives. Health outcomes are clearly socially patterned with greater burden related to lower socioeconomic status (SES) as well as ethnic/minority group membership. Lower SES, ethnic minority group status, and residence in a more toxic environment are closely intertwined in the United States (US). Among low-SES areas, those with predominantly minority, segregated populations seem especially burdened. In addition, studies in urban environments show geographic variation in health outcomes across large cities and neighborhoods within cities that cannot be explained by economic factors alone. Although available data are more limited, studies in rural areas also suggest the stratification of risk based on SES and the proportion of minorities. The principles of social and environmental justice are thus inherently linked to health. Populations of lower socioeconomic position are disproportionately exposed to chemical stressors including irritants (e.g., tobacco smoke), pollutants (e.g., diesel-related particles), and housing-related toxicants (e.g., indoor allergens). Moreover, individuals may also live in families and communities that are differentially burdened with social toxins (e.g., crime or interpersonal violence exposure, discrimination, financial strain), which, in turn, may be related to the variable experiencing of psychosocial stress. While far more attention has been given to physical environmental toxicants, more recent consensus statements by the National Academies of Science (2) and the National Institutes of Health (3) point to the need to consider social environmental and behavioral factors as well. Indeed, non-chemical stressors (i.e., social determinants and psychological stress) may be as toxic as chemical pollutants in the environment (4).

**Figure 1:**
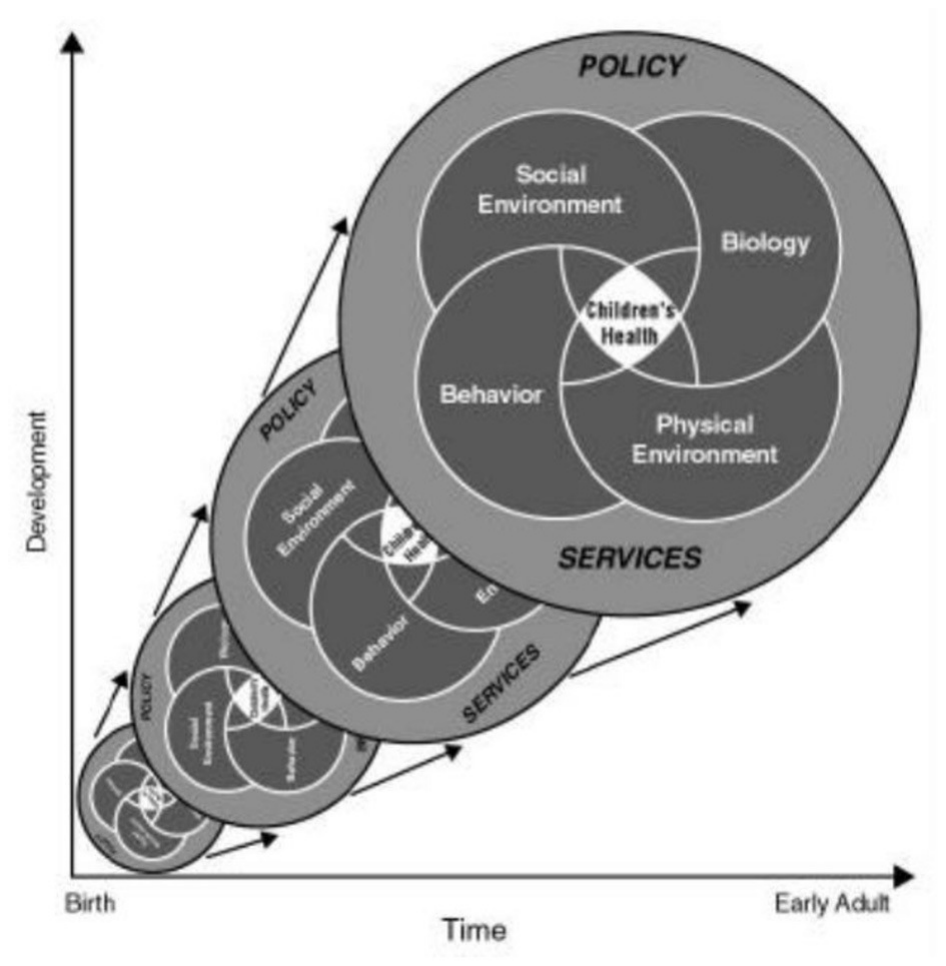
Characterizing environment across the life course: an ecological approach (http://www.ncbi.nlm.nih.gov/books/NBK92206/). Policy and services affect the social environment, biology, behavior, and physical environment, which in turn affect children's health. These factors change across time (x-axis) and development (y-axis), influencing children's health along the way.

**Figure 2:**
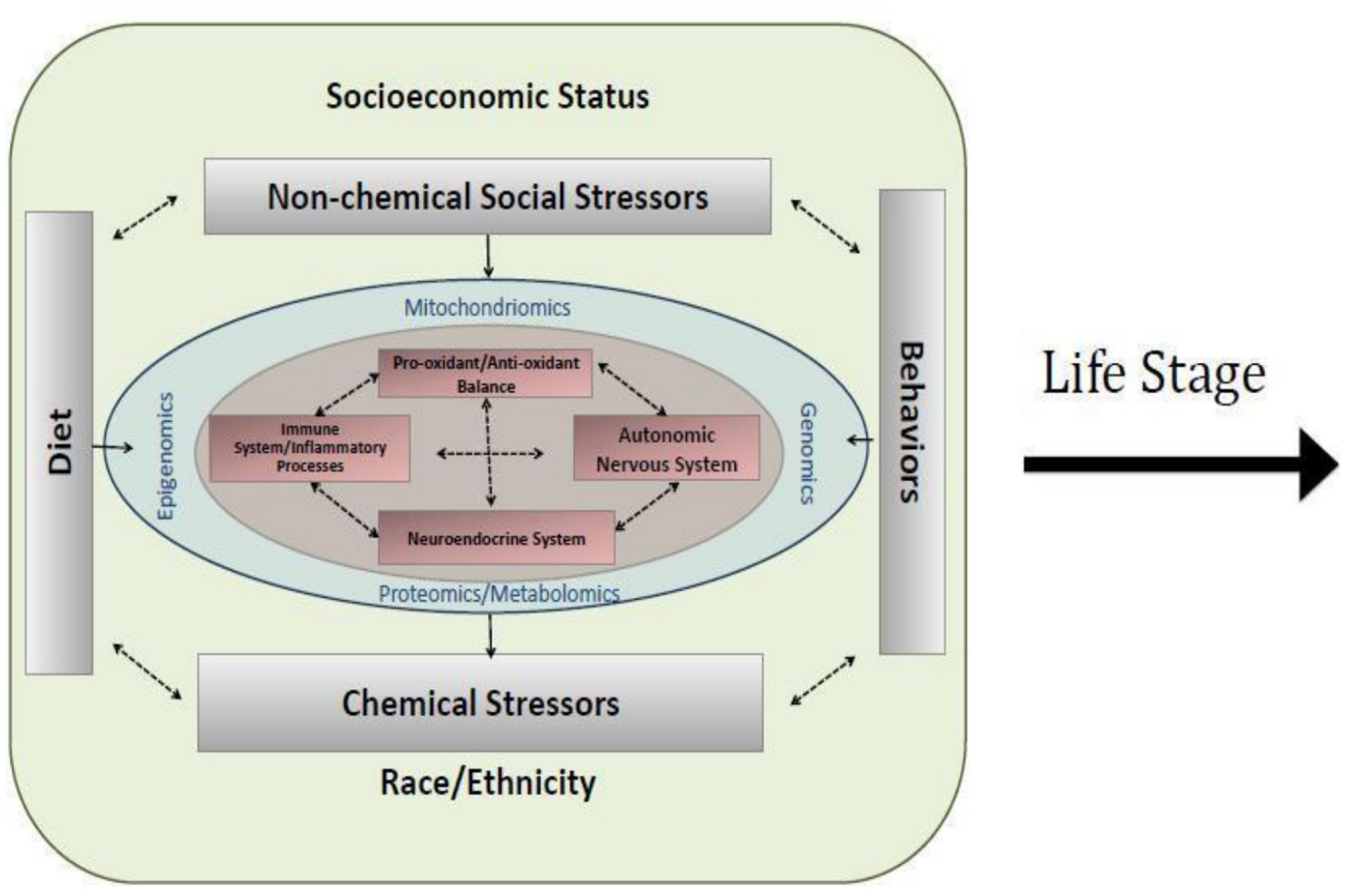
Conceptualization of a cross-section of the exposome within a life stage interval. Socioeconomic status, non-chemical stressors, race/ethnicity, chemical stressors, diet, and behavior interact with genomics, epigenomics, mitochondiomics, and proteomics/metabolomics to influence healthy biological functioning, including oxidant/antioxidant balance, autonomic nervous system, inflammation, and neuroendocrine functioning.

As depicted in Figure 1, the proposed approach to assessing environmental risk in the NCS took a multi-level approach that explicitly recognized the embedding of health risk within its biologic, socioeconomic, social, and physical environmental as well as community, and societal contexts (5). Such an approach may help to explain heterogeneities in disease expression across development as well as socioeconomic and geographic boundaries. This framework also incorporated the interdisciplinary life course framework that expands on the developmental origins view and emphasizes the physical, biological, and social/behavioral factors throughout life that independently, cumulatively, and interactively influence health and development (3, 6). The conceptualization of the exposome accommodates this recognized complexity and provided the overarching organizational framework that guided recommendations for environmental assessments in the NCS. The exposome considers multiple exposures (e.g., social and physical) and behaviors that may influence exposure to environmental factors (e.g., diet, smoking, drug use, use of consumer products, parenting styles), as well as consequences of environmental exposures indexed via biomarkers (e.g., epigenetic changes and/or associated biological responses or endogenous processes such as immunomodulation or oxidative stress) and behavioral constructs (e.g., emotion regulation, temperament, attributional style) to more comprehensively characterize the impact of environmental exposures throughout the lifespan (see Figure 2).

Figure 2 also depicts key regulatory systems (immune system, neuroendocrine system, autonomic nervous system, and pro-oxidant/anti- oxidant balance) that may be influenced by environmental factors and consequently program health trajectories. While mechanisms underlying programming effects of the wide array of environmental factors thought to contribute to health and development are not completely elucidated, banking biospecimens in critical windows of development to allow more comprehensive approaches to understanding networks and mechanisms that may be involved is critical. For the NCS, this included taking advantage of evolving high-throughput “omics” approaches, some of which are depicted in Figure 2 (epigenomics, metabolomics, mitochoriomics, microbiomics). For example, epigenetics, the study of changes in gene expression without change in DNA sequence, has emerged as a leading mechanism through which environment may impact health and development in a dynamic way over the life course. A growing number of studies have demonstrated associations between DNA methylation, a leading epigenetic mechanism, and exposures to a range of chemical and non-chemical environmental stressors. While much has been documented on the importance of environmentally induced epigenetic changes for programming health in early development (prenatal, early childhood) (2, 7, 8), this is an important mechanism to understand across developmental periods, for example, recent evidence suggests a role over a range of developmental windows in relation to pubertal timing (9). Microbiomics, the study of changes in the oral and gut microbiome, have been increasingly associated with metabolic changes and diseases such as cardiovascular, gastrointestinal, and pancreatic cancer risks. For example, in healthy mouths, the oral microbiome is dominated by *Firmicutes, Bacteroidetes, Proteobacteria, Actinobacteria, Spirochaetes*, and *Fusobacteria*; however, in oral diseases, *lactobacilli* and *Streptococcus mutans* produce excessive amounts of acid to produce dental cavities. *P. catoniae* and *N. flavescens* were associated with cavity-free mouths. Bacterial communities can be determined from biospecimens using pyrosequencing or 16SrDNA metagenomic analysis of buccal cells and fecal samples. The interplay with mithochondrial processes has emerged more recently in relation to environmental influences on health and development (10). While “omics” approaches are more extensively discussed by the Physical Health & Systems Domain, we briefly introduce these concepts here to exemplify their central importance to the concept of measuring the exposome to study environmental influences on health and development in the NCS.

Conceptually, the framework needs to account for a reasonably comprehensive spectrum of exposures, timing and duration of exposures, and acute and chronic responses to the exposures (e.g., behavioral, physiologic) over time. In a longitudinal study design, this will allow researchers to ask, “Where are impacts and responses occurring? Is it at the cellular, the genetic, or the organ, individual, household, specific population group, or community level?” Indeed, the answers are unlikely to be exclusive.

### Need for a Developmental Framework

Effects to children can occur from exposures at various stages in the life course, including preconception. Effects on either the sperm or egg prior to conception have been known to impact development and, in many cases, overall viability of the conceptus. Recent emphasis on heritable epigenetic changes that occur via either germ cell has advanced not only the concept of male-mediated developmental toxicity but also illustrated a myriad of non-coding RNA mechanisms including maternal inheritance. In the male, both progenitor and replenishing spermatogonial cells have allowed the male to repair and fertility to return in some cases. However, high doses of environmental agents can also destroy male reproductive capability beyond repair. It is also becoming increasingly apparent that certain phenotypes are inherited across generations independent of the information contained in the DNA sequence, by factors in germ cells that remain largely uncharacterized (11). Cohort studies should attempt to collect information concerning the exposures to the grandparents and parents, particularly in their own childhood.

Plasticity, the ability of the organism to respond to an insult and still maintain homeostasis, can be overcome, and health impacts can arise as a consequence of environmental exposures during critical life periods affecting key physiological systems that operate in orchestrating underlying developmental processes (12). The concept that factors other than genetic susceptibility act early in life to permanently organize or imprint physiological systems is known as perinatal programming and has formed the basis of the developmental origins of disease research. The NCS pregnancy cohort will allow us to examine the implications of a number of environmental exposures in this vulnerable period of development. Prenatally, children are particularly vulnerable to disruption of developmental processes during relatively narrow time windows of development. Exposure to environmental toxicants during prenatal and/or early postnatal development may alter the normal course of morphogenesis and maturation, resulting in changes that affect both structure and function of multiple organ systems, e.g., the respiratory and neurological systems (13). When normal development is altered, the early effects may persist into adult life, magnifying the public health impact.

Although the range of health outcomes that may ultimately be of interest may have disparate vulnerability factors and natural histories (e.g., many do not manifest until later childhood or even into adulthood), most will have their roots very early in development, including prenatally—a notion grounded in the “developmental origins of chronic disease hypothesis (12, 14, 15).” Studies suggest that characteristics of the *in utero* environment, independent of genetic susceptibility, influence embryonic and fetal development, including neurological, endocrine, cardiovascular, respiratory, and immune development. Early misconceptions that organ systems finished their overall development at the end of the second trimester gave rise to the inaccurate concept that exposure to factors such as alcohol after this time would not result in developmental toxicity. We now know that these patterns are much more complex and the windows of vulnerability much larger. Specific biological events occur in each trimester of pregnancy, changing susceptibility to environmental factors across time, agent, and dose. Since scientists demonstrated that environmental impacts during the second trimester could result in specific birth defects (16), they recognized that when the patterns of proliferation and differentiation were tracked, a prediction of specific windows of susceptibility was evident.

Many other concepts of developmental toxicology are known for this period. The developing fetus and young child are uniquely vulnerable because their respiratory, immune, neuroendocrine, and xenobiotic detoxification systems are in the process of development throughout pregnancy and early childhood (17, 18). For example, we know the conceptus *in utero*, and young children differentially gain the ability to metabolize environmental chemicals; therefore, knowing which metabolizing enzymes are present at various times *in utero* and post-natally is significant in understanding when and how chemicals impact development. Such knowledge can identify the form and potential for chemicals to impact development. For example, this information is used clinically to treat the neonate when drug metabolism activity levels exceed adult human levels of metabolism (requiring larger-than-adult drug doses for some clinical treatments). This means that differential times of exposure to the same toxicant are in reality, very different. For the NCS, we need to ensure that sufficient assessments and biospecimens are available to support these observations and interpret intrauterine exposures.

Adolescence represents another key transitional period in the life course that is vulnerable to environmental programming (19–22). The adolescent period corresponds to the attainment of adult height and sexual maturity. Key homeostatic systems are going through changes during the process of sexual maturation, involving complex interactions between the central nervous system (CNS), hormone-secreting organs, and immune function, all of which can be affected by environmental factors (23, 24). The period from adolescence through early adulthood may also be a time of susceptibility to environmental toxins/toxicants because organ systems (e.g., the lung, brain) are in the final phase of growth and maturation, and because adolescence is a period of rapid social and emotional change.

The NCS process was also guided by a life course approach, which considers both the more immediate and longer-term effects of physical and social exposures during all periods of development (e.g., gestation, childhood, adolescence, early adulthood, and later adult life) (3). Recommended age-appropriate measures and timing of assessments were guided by our understanding of differential vulnerabilities to disease between individuals depending on the developmental stage when exposure occurs (e.g., childhood, adolescence, young adulthood, and later adult life). Moreover, adverse exposures during critical periods may have concurrent effects, latent effects (not observed for a number of years) and cumulative effects (adding up as subjects age) that negatively impact health outcomes and trajectories over the life course (25). Chronic exposures recurring or persisting over multiple developmental periods should also be considered (cumulative chemical exposures, ongoing traumas, persistent poverty, etc.).

**Figure 3:**
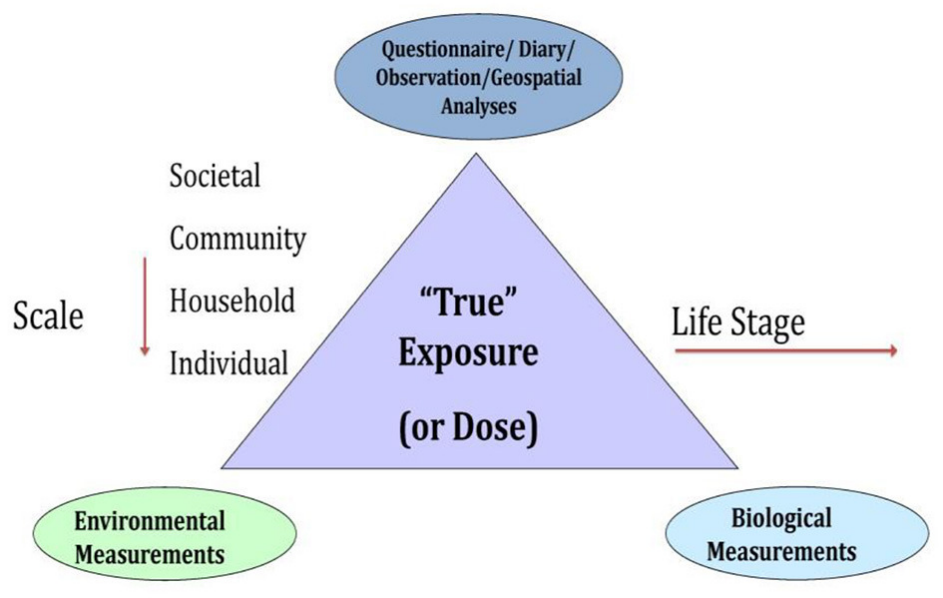
Combined approaches for the National Children's Study environmental exposure assessment.

A key challenge was, thus, to identify where, when, and what measures, environmental samples, and biospecimens should be collected to characterize potential exposure factors and effects of exposures across the life course in a reasonably comprehensive manner. External exposure assessment relies on measuring factors broadly characterized, such as chemical and non-chemical stressors, diet, and behaviors (Figure 2). Moreover, the built environment influences each of these exposure classifications. Combined approaches, some overlapping, including survey measures, observational measures, direct monitoring approaches with laboratory analysis, and geospatial analysis leveraging extant data gathered for other purposes, can be used to characterize the environment (see Figure 3, and summarized below). It is important to identify at what level of biological organization and life stage to collect such assessments. Figure 3 illustrates the need to look at this at various ecological levels, for example, at the individual, family, household, and community level in order to analyze and interpret environmental profiles.

## Overview

The National Children's Study (NCS), Health Measurement Network (HMN), and Environmental Domain Working Group addressed the need for an environmental measurement framework to answer critical questions regarding children's health by developing recommendations for assessment of environmental exposures across the life stage within the NCS. The attached grid (Tables 1–6) summarizes the group's recommendations of measurement for the following five domains:

1. *Chemical and biological exposures* including organic chemicals (pesticides, organochlorine pesticides, organophosphate pesticides and metabolites, carbamate pesticides, fungicides, herbicides, brominated flame retardants, disinfection byproducts, environmental tobacco smoke markers, environmental phenols, parabens, perfluorinated compounds, bisphenol A and phthalates, polychlorinated biphenyls, polycyclic aromatic hydrocarbon and metabolites, and volatile organic hydrocarbons), inorganic chemicals (metals, and perchlorate and other anions), and biological exposures (allergens and mold, and microbes);
2. *Physical environment exposures including* community design, physical safety, access to food resources, radiation exposure, ultraviolet radiation, radon, and noise exposure;
3. *Stress including social support and coping*;
4. *Social determinants of health* including socioeconomic status, family relationship quality, parent/caregiver mental health, parenting difficulties, attachment, child care/school characteristics, community violence, social capital, school environment, peers and neighborhood, and mentoring; and
5. *Modifying factors* including diet, obesity, and physical activity. Sources of information collected in the Grid are summarized below.

**Table 1:** Domain: Chemical exposures.

**NCS Measurement category/domain**	**Subdomain**	**Data collection method**	**Recommended measure**	**Time/burden/cost**	**Status of recommended measure**	**Notes**	**Visit Type**
Health behaviors/diet/chemicals	Environmental contaminants through food (metals, mercury, SVOCs, PCBs, etc.)	SAQ	Automated Self-Administered 24-h Recall (ASA24)	30–45 min/$	Ready		Core (M, C)
		SAQ	NCS Dietary FFQ (SAQ)—short survey	10 min/$	Ready		Core (M, C)
		SAQ	NCS 6-month infant feeding SAQ; NCS 12-month Child SAQ (food items)	4–7 min/$	Ready		Core (M, C)
		Biospecimens	Breast milk	13 min/$$	Ready	Collect as long as child breast feeds (better to measure child's biospecimens)	PHS (M)
		Sample	Formula	7–13 min/$$	Ready	Collect as long as child uses formula, Better to measure in child's biospecimens if possible, Could be self-collected	In-home
Physical environment/chemical	Personal product use	Questionnaire	NCS Initial Vanguard Study −12, 18, 24 months Child Instrument	2 items (1 min)/$	Ready	Focused on insecticides, lice treatment	Parent Remote
	Personal product use	Questionnaire	NCS	3 min $	Ready		Parent Remote
	Personal product use	Questionnaire	TBD	3 min $	TBD	Need questionnaire for older ages	Child Remote
	Organics: organochlorine pesticides, organophosphate pesticides, carbamate pesticides, fungicides, herbicides, disinfection byproducts, environmental phenols, parabens, phthalates, PAHs and metabolites, phytoestrogens, VOCs Inorganics: perchlorate and other anions	Biospecimen	Urine	7–13 min/$$	Ready		PHS
	Organics: BFRs, ETS, PCBs, PFCs	Biospecimen	Blood—Serum	11–17 min/$$	Ready		PHS
	Organics: disinfection byproducts, PFCs, PAHs and metabolites	Biospecimen	Blood—Venous, DBS	11–17 min/$$	DBS needs development		PHS
	Organics: environmental phenols, phthalates	Biospecimen	Teeth	$$	Needs development		PHS
	Metals (lead, manganese, mercury, strontium)	Biospecimens		$$	Ready		PHS
	Metals (cadmium, lead, manganese, inorganic mercury, total selenium)	Biospecimens	Blood	11–17 min/$$	Ready		PHS
	Metals (cadmium, mercury, lead)	Biospecimens	Placenta	5 min/$$	Ready		PHS
	Metals (arsenic, cadmium, chromium, copper, iron, manganese, methyl mercury, nickel, lead, strontium)	Biospecimens	Hair	6 min/$$	Ready		PHS
	Metals (arsenic, cadmium, lead, manganese, nickel)	Biospecimens	Nails	6 min/$$	Ready		PHS
	Metals (arsenic, cadmium, lead, mercury)	Biospecimens	Breast milk	13 min/$$	Ready		PHS
Physical environment/chemical/non-residential	Occupational/hobby: parent—pre-pregnancy, pregnancy, early childhood	Questionnaire	NCS Initial Vanguard Study—Occupation and Hobbies (Pregnancy visits)	10 min/$	Needs development	To include specific hobby questions	Parent Remote
	Occupational/hobby: child	Questionnaire	Occupation—Child	5 min/$	TBD	Administered for each child job/activity	Child Remote
	Commuting exposures	Extant data/GIS	ADDRESSES	Limited burden/Variable $	Ready	GIS to link to air pollution, traffic, etc. to addresses (see physical env below)	GIS, P,C
	Commuting exposures, parent	Questionnaire	NCS Initial Vanguard Study—Commuting (T1 Mother), M4.2—Commuting (PV1)	4 items (2 min) $	Ready		Core
	Commuting exposures, child	Questionnaire	TBD	4 items (2 min) $	TBD		Child Remote
Physical environment/chemical/residential	Occupational: take-home exposures	Questionnaire	NCS, M4.0/M4.1 Occupational Exposures 36, 48, 60 M (Take Home)	9 items (4 min) $	Ready	Focus on toxicants that cannot be measured in biospecimens Annual (about HH)	Parent Remote
	Allergens, endotoxins, molds	Vacuum bag dust sampling	NCS—Bulk Dust, Dust From Vacuum Cleaner or Collected Sample	15 (14–25) min/$$	Ready	Self-collect wipe/bag sample	In Home
	SVOCs, pesticides				Ready	Can be participant collected	
	SVOCs, pesticides	Dust wipe sampling	NCS DC Collect	7 min/$$	Ready	Can be participant collected (would need development)	In Home
	Metals/inorganics	Dust wipe sampling	NCS	7 min $$	Needs development	For self-collected samples	In Home
	Indoor air—particulate matter, PAHs, carbon, metals	Indoor air sampling	NCS Initial Vanguard Study (Revised)	20 min $$	TBD	Research in progress for alternate device Cannot get from biospecimens	In Home
	Indoor air—CO	Indoor air sampling		5 min $$	TBD	Can get from biospecimens	In Home
	Indoor air—NOX	Indoor air sampling	NCS—Badge	5 min $$	Ready	Can be participant collected Cannot get from biospecimens	In Home
	Indoor air—O_3_	Indoor air sampling	NCS—Badge	5 min $$	Ready	Trigger sampling - not many triggers, Can be participant collected, Cannot get from biospecimens	In Home
	VOCs	Indoor air sampling	NCS—Badge	5 min $$	Ready	Samples cannot be stored, Cannot easily get from biospecimens	In Home
	Carbonyls	Indoor air sampling	NCS – badge	5 min $$	Ready	Samples cannot be stored, Cannot get from biospecimens	In Home
	Pesticides	Water sampling	NCS	5 min/$$	Needs development	Can be participant collected	In Home
	Pharmaceuticals	Water sampling	NCS	5 min/$$	Needs development	Can be participant collected	In Home
	Carbonyls	Observation	NCS Dwelling Unit Observations/Sources in Home	15 min $	Ready	Every move	In Home
	Smoking in home	Observations	NCS Dwelling Unit Observations/Sources in Home		Ready		
	Indoor air—particulate matter, PAHs, CO	Observations	NCS Dwelling Unit Observations/Sources in home		Ready		
	Indoor air—NOX	Observations	NCS Dwelling Unit Observations/Sources in Home		Ready		
	Indoor air—O_3_	Observations	NCS Dwelling Unit Observations/Sources in Home		Ready		
	Metals: arsenic, lead	Observations	NCS Dwelling Unit Observations/Sources in Home		Ready		
	Housing characteristics	Observations	NCS Dwelling Unit Observations/Sources in Home		Ready		
	Allergens, mold	Observations—sources in home	NCS Dwelling Unit Observations/Sources in home		Ready		
	Structural characteristics	Observations	NCS Outdoor Structural Observations	15 min $	Ready	Every move	External
	Grounds	Observations	NCS Outdoor Structural Observations		Ready		
Physical environment/residential	Housing characteristics	Questionnaire	NCS, M4.0 - Core Household	19 items (9 min) $	Ready	Based on American Healthy Homes Survey (minimum every move)	Core
	Interior home safety	Observation	NCS Dwelling Unit Observations/Sources in Home	As above	Ready	Every move	In Home
	Exterior home safety	Observation	NCS Outdoor Structural Observations	As above	Ready	Every move	External
	Access to guns	Observation	NCS Dwelling Unit Observations/Sources in Home	As above	Ready	Every move	In Home

*The column Visit Type maps the assessment to data collections that in some cases are mapped to other domains such as Physical Health Systems (PHS) and Social–Emotional–Behavioral (SEB) (see respective chapters in this edition)*.

*$, inexpensive; $$, expensive; BFRs, brominated flame retardants; C, child; CO, carbon monoxide; DBS, dried blood spots; DC, data collector; ETS, environmental tobacco smoke; FFQ, food frequency questionnaire; GIS, geographic information system; M, mother; NOx, nitrogen oxides; O3, ozone; PAHs, polycyclic aromatic hydrocarbons; PCBs, polychlorinated biphenyls; PFCs, perfluorinated compounds; SAQ, self-administered questionnaire; SVOCs, semi volatile organic compounds; TBD, to be developed; VOCs, volatile organic compounds*.

**Table 2:** Domain: Physical environment.

**NCS measurement category/domain**	**Subdomain**	**Data collection method**	**Recommended measure**	**Time/burden/cost**	**Status of recommended measure**	**Notes**	**Visit Type**
Physical environment/chemical	Physical location—home, school, work	Questionnaire	ADDRESSES	$	Ready	Throughout, as changes Need for GIS links	GIS
Physical environment/residential	Home safety	Observation	NCS	$	Ready	See above—in home obs	NA
	Access to guns	Observation	NCS	$	Ready	See above—in home obs	NA
	Noise	Sampling—area	In home monitoring device (e.g., dosimeter)	15 min $$	Ready	7 days	In home
Physical environment/community	School/child care characteristics—physical	Questionnaire	TBD	10 items, 5 min $	TBD	Every new school/child care	PHS
Physical environment	Noise, home	Questionnaire	Noise sources, annoyance	5 min $	Ready	In-person if noise monitor set up	Parent remote
	Noise, home	Sampling—personal	Personal monitoring device such as dosimeter	15 min $$	TBD		Child in-person
	Noise, young child	Questionnaire	TBD—noisy activities, occupational as child ages	5 min $	TBD		Parent remote
	Noise, older child	Questionnaire	TBD—noisy activities, occupational as child ages	5 min $	TBD		Child remote
	Electromagnetic fields (EMF)		None		TBD	Difficult to measure Highly variable	NA
	Radon	Extant data/GIS	ADDRESSES	Limited burden/variable costs	Ready	Could also use direct canister measure	GIS
Health behaviors	Sun exposure	Questionnaire			TBD		Parent remote
	Sun exposure	Extant data/GIS	ADDRESSES	Limited burden/variable costs	Ready		GIS
	Sun exposure (UV)	Biospecimen	Urine (thymine dimer)	$$	TBD		PHS
	Sun exposure (UV)	Biospecimen	Blood (vitamin D)	$$	Ready		PHS
	Media use	Questionnaire			TBD		PHS
Social environment/community	Neighborhood crime	Extant data/GIS	ADDRESSES	Limited burden/variable costs	Ready		GIS
	Transportation corridors and terminals	Extant data/GIS	ADDRESSES	Limited burden/variable costs	Ready	These could all be observation as well	GIS
	Commercial structures	Extant data/GIS	ADDRESSES	Limited burden/variable costs	Ready		GIS
	Greenspace	Extant data/GIS	ADDRESSES	Limited burden/variable costs	Ready		GIS
	Food access (food swamps, food deserts, etc.)	Extant data/GIS	ADDRESSES	limited burden/variable costs	Ready		GIS
	Housing damage	Extant data/GIS	ADDRESSES	Limited burden/variable costs	Ready		GIS
	Property disorder	Extant data/GIS	ADDRESSES	Limited burden/variable costs	Ready		GIS
	Vacancy	Extant data/GIS	ADDRESSES	Limited burden/variable costs	Ready		GIS
	Segregation	Extant data/GIS	ADDRESSES	Limited burden/variable costs	Ready		GIS
	Neighborhood characteristics—physical	Observation	NCS	$	Needs development	To assess factors not obtained from extant data/GIS, every move	External
	Neighborhood characteristics—perception	Questionnaire	NCS—participant perception	11 items, non-proprietary	Ready	Depends on characteristic Every move, or annually	Core
	Child care characteristics—social		Based on NICHD Early Child Care Study		TBD	Every new care giver	SEB

*The column Visit Type maps the assessment to data collections that in some cases are mapped to other domains such as Physical Health Systems (PHS) and Social–Emotional–Behavioral (SEB) (see respective chapters in this edition)*.

*$, inexpensive; $$, expensive; C, child; GIS, geographic information system; M, mother; TBD, to be developed*.

**Table 3:** Domain: Stress.

**NCS measurement category/domain**	**Subdomain**	**Data collection method**	**Recommended measure**	**Time/burden/cost**	**Status of recommended measure**	**Notes**	**Visit Type**
Psychosocial/emotion/stress and coping	Maternal stress/trauma (life course)	Questionnaire	Life Stressor Checklist—Revised (26)	30 items (15 min), non-proprietary	Ready	Each contact	Parent Remote
	Maternal chronic stress	Extant data/GIS	ADDRESSES	Limited burden/variable costs	Ready		GIS
	Childhood stress/trauma (life course)	Questionnaire	Traumatic Event Screening Inventory—Parent Report Revised (TESI-PRR) (27, 28)	25 items (12 min) Non-proprietary	Ready	Maternal report child 0–6 years mother answers about kid	Parent Remote
	Perceived stress	Questionnaire	NIH toolbox Perceived Stress Scale (PSS) 4- or 10-item, plus parenting modification PROMIS Pediatric Stress Response, psychological stress short form (29)	4 items, non-proprietary	Ready	Each contact	SEB
	Maternal Coping	Questionnaire	Brief Coping Orientation to Problems Experienced (COPE)	28 items (14 min) Non-proprietary	Needs development	Look into reducing–match to negative life event	Parent Remote
	Caregiver/child stress/trauma (life course)	Biospecimens	Hair/salivary cortisol	14 min $$	Ready	Possible self-collect	PHS
Psychosocial/parenting	Caregiver stress/trauma (life course)	Questionnaire	Childhood Trauma Questionnaire—short form (CTQ-SF) (30)	12 Items (6 min), non-proprietary	Ready	Asked of mother once in prenatal evaluation (target mother)	Parent Remote
Psychosocial/mental health	Pregnancy-related Anxiety	Questionnaire	Pregnancy Anxiety Scale (PAS) (31)	7 items (3 min), non-proprietary			Parent Remote
	Depression	Questionnaire	Edinburgh Postnatal Depression Scale (EPDS) (32)	10 items, non-proprietary	Ready	Use SEB measure	SEB
Social environment/community	Maternal stress/trauma (life course)—community violence—parent	Questionnaire	Abbreviated Exposure to Community Violence (ETV) Survey (33)	5 items (3 min), non-proprietary	Ready	Maternal report on their experience and child under 8 years, Annual	Parent Remote
	Maternal stress/trauma (life course)—community violence—child	Questionnaire	Abbreviated Exposure to Community Violence (ETV) Survey (33)	5 items (3 min), non-proprietary	Ready	Maternal report on their experience and child <8 years, Annual	Child Remote
Social environment/life experiences	Negative life events, parent	Questionnaire	Current Crisis in Family Systems (CRISYS life) events scale (34)	64 items (10–20 min), non-proprietary	Ready	Annual - once in first year and then every 2 years to age 5 then every 3 years	Parent Remote
	Negative life events, child	Questionnaire	Current Crisis in Family Systems (CRISYS life) events scale (34)	64 items (10–20 min), non-proprietary	Ready	Annual - once in first year and then every 2 years to age 5 then every 3 years	Child Remote
Social support	Interpersonal support	Questionnaire	PROMIS SF self-report: Informational support, Instrumental support, Emotional support, Companionship [or Interpersonal Support and Evaluation List (ISEL)] (29)	PROMIS (4 items ea) ISEL (12 items)	Ready	Annual	SEB
Social environment/social support	Social capital	Questionnaire	Social capital from the General Social Survey, or 10-item perceived stress scale (NIH toolbox)	4 items (2 min), non-proprietary	Ready		Parent Remote
Social environment/social support	Social capital	Questionnaire	Community Social Capital (from Health Behavior in School-Aged Children): SEB (35)	5 items	Needs development		SEB

*The column Visit Type maps the assessment to data collections that in some cases are mapped to other domains such as Physical Health Systems (PHS) and Social–Emotional–Behavioral (SEB) (see respective chapters in this edition)*.

*$, inexpensive; $$, expensive; C, child; GIS, geographic information system; M, mother; TBD, to be developed*.

**Table 4:** Domain: Social determinants.

**NCS measurement category/domain**	**Subdomain**	**Data collection method**	**Recommended measure**	**Time/burden/cost**	**Status of recommended measure**	**Notes**	**Visit Type**
Demographics	SES	Questionnaire	MacArthur Foundation's Network on Socioeconomic Status; Health's Sociodemographic Questionnaire (36)	Non-proprietary	Ready	Look at NCS SES Qx	Core
Parenting	Parenting difficulties	Questionnaire	Parenting Stress Index (PSI) (37)	11 items, non-proprietary	Ready		SEB
	Parenting self-efficacy	Questionnaire	Parenting Sense of Competency Scale (38)	7 items	Ready		SEB
	Parenting style	Questionnaire	Parenting style and dimensions Qx (39–42)	32 items	Ready		SEB
Social environment/social support	Family relationship quality	Questionnaire	Brody Parenting Scale (parent and/or child) (43)	23 items, non-proprietary	Ready	Annual	SEB
	Family relationship quality	Questionnaire	Security in the Interparental Subsystem Scale (SIS) child (44)	13 items, non-proprietary	Ready	Annual	SEB
	Family relationship quality	Questionnaire	Family Routines Inventory (FRI)—reduced Also Family Belonging, Family Involvement, Couples Satisfaction Index, Conflict and Problem Solving; Nursing Satellite Assessment Teaching Scale (NCATS); Berkeley Puppet interview (45)	Non-proprietary	Ready	Annual	SEB
	Racial experiences/discrimination	Questionnaire	Experiences of Discrimination (EOD) Scale, or David Williams—Everyday Discrimination Scale (1997) (45, 46)	Non-proprietary	Ready	Annual—m, f, c	SEB
	Marital/close relationships (life course)	Questionnaire	Perceived Relationship Quality Components (PRQC) Inventory, or Marital –Brief 6-item marriage/close Rx Qx (Fletcher) (47)	Non-proprietary	Ready		SEB
	Teacher connectedness/student engagement	Questionnaire	Sense of School as Community Scale (48)	20 items, 10 min (?) Non-proprietary	Ready	Best if done toward end of school year	Parent Remote
	Peer/romantic relationships for child		PROMIS Peer Relationship measure	Non-proprietary	Ready		SEB
	Peer relationships for child	Questionnaire	PROMIS Pediatric Peer Rx—short form	Non-proprietary	Ready	Annual	SEB
	Peer relationships for child	Questionnaire	Revised Peer Experiences Questionnaire (RPEQ)	Non-proprietary	Ready	Annual Gets at bullying	SEB
	Peer relationships for child	Questionnaire	Peer Pressure Inventory	Non-proprietary	Ready	Annual	SEB
	Peer relationships for child	Other SEB	ITSEA Prosocial Peer Relations Scale; NIH Toolbox Social Withdrawal Survey, Proxy Positive Peer Interaction Survey, Proxy Peer Rejection Survey, Pediatric Loneliness, Pediatric Friendship, Perceived Hostility, Perceived Rejection				SEB
Social environment/community	Acculturation		NONE		TBD	Surrogates—language, country of origin	TBD
	Cultural values/ethnic identity		NONE				TBD
	Religiosity/spirituality	Questionnaire	Duke University Religion Index (DUREL) Hispanic SOL study (49)	5 items, non-proprietary	Ready	Annual	SEB
	Access to medical care	Questionnaire	NCS	$	Ready		SEB
Social environment/financial resources/insurance	Health insurance	Questionnaire	NCS	$	Ready		SEB
	Food security/housing stability (move frequency)	Questionnaire	ADDRESSES	$	Ready	Standard data collection—address changes	GIS
Social environment/academic experience	School attendance	Extant data/GIS	ADDRESSES	Limited burden/variable costs	Ready		GIS
	Academic performance/achievement	Questionnaire		10 items, 5 min	NA/TBD	Annual after 6 years	Parent Remote
	Academic performance/achievement	Extant data	School records		NA/TBD		GIS
Social environment/recreational activities	Extracurricular activities	NA	NONE		NA	Not considered high priority	NA
Psychosocial/emotion	Intrapersonal resources (self-esteem, optimism)	Questionnaire	Rosenberg Self-Esteem Scales (50)	10 items, Non-proprietary	Ready	Annual	SEB
	Intrapersonal resources (self-esteem, optimism)	Questionnaire	Life Orientation Test (LOT-R) (51)	6 items, Non-proprietary	Ready	Annual	SEB
	Intrapersonal resources (self-esteem, optimism)	Questionnaire	Primary Care Evaluation of Mental Disorders (PRIME-MD)	Non-proprietary	Ready		SEB
	Temperament	Questionnaire			Ready		SEB
Psychosocial/mental health	Mental health of mom and dad (e.g., depression, hostility, intrapersonal resources)	Questionnaire	Breslau 7-item PTSD Screener, or PROMIS Adult Mental Health items (Depression, Anger, Anxiety) (52)	7 items, non-proprietary	Ready		SEB
	Psychological well-being	Questionnaire	Carol Ryff; Psychological Well Being Scale (RPWB) (53)	Non-proprietary	Ready	Annual	SEB
	Child mental health (behavior problems, depression)	Questionnaire	Child Behavior Checklist (CBCL), or NIH Toolbox/PROMIS Anger (3–7, 8–17), Sadness (3–7) Bullying Qx, Peer Pressure, School Belonging/Sense of School as a Community, every year	Non-proprietary	Ready		SEB
Psychosocial/behavior	Externalizing behavior (aggression, antisocial)	Questionnaire					SEB
Psychosocial/parenting	Parenting difficulties	Questionnaire	Parenting Stress Index (PSI)	11 items, non-proprietary	Ready		SEB
	Maternal sensitivity	Questionnaire					SEB
Psychosocial/stress and coping	Coping—adolescent	Questionnaire	The Responses to Stress Questionnaire (RSQ) for adolescents (54)	58 item/29 min (?), non-proprietary	Ready		Child Remote
Psychosocial/emotion	Resilience						SEB
Health behaviors/substance use	Substance use	Questionnaire				Both SEB and PHS report this measure	SEB/PHS
Health behaviors	Child health behaviors						SEB

*The column Visit Type maps the assessment to data collections that in some cases are mapped to other domains such as Physical Health Systems (PHS) and Social–Emotional–Behavioral (SEB) (see respective chapters in this edition)*.

*$, inexpensive; $$, expensive; C, child; GIS, geographic information system; M, mother; TBD, to be developed*.

**Table 5:** Domain: Modifying factors.

**NCS Measurement category/domain**	**Subdomain**	**Data collection method**	**Recommended measure**	**Time/burden/cost**	**Status of recommended measure**	**Notes**	**Visit Type**
Demographics	Race ethnicity	Questionnaire		$	Ready		Core
Physical health/current health status	Height	Measurement		$$	Ready		PHS
	Weight	Measurement		$$	Ready		PHS
Health behaviors/diet	Nutritional status	SAQ	Automated Self-administered 24-h Recall (ASA24) system (55)	30–45 min $	Ready	Used to validate FFQ, does not need to be done for everyone, mobile version, photos of meals, week day, weekend	Core
	Nutritional status	SAQ	NCS 6 months infant feeding SAQ; NCS 12 months Child SAQ (food items)	4–7 min $	Ready	12 months—breast and formula feeding, first fed specific types of other foods (cow's milk, pureed food, “adult foods,” etc.); Q28 on supplements	Core
	Nutritional Status	SAQ	NCS Dietary Food Frequency (FFQ) SAQ-short survey	10 min $	Ready	Fish, organic questions limited; Positive answer could trigger additional module, biospecimen analysis	Core
Physical health/medication use	Nutritional status through supplements	Questionnaire	NCS Core QX–Over the counter medications (prescribed medication information is obtained through PHS)	$	Ready		PHS
Physical health/biologic	Nutritional status Antioxidants (vitamins A, E); EFAs, etc.	Biospecimens	Blood	$$	Ready	Recommended measure for contaminants, where applicable—may not be able to get for pre-conception	PHS
Health behaviors/physical activity	Physical activity	Monitor	NCS accelerometer/GPS	$$	Ready		SEB

*The column Visit Type maps the assessment to data collections that in some cases are mapped to other domains such as Physical Health Systems (PHS) and Social–Emotional–Behavioral (SEB) (see respective chapters in this edition)*.

*$, inexpensive; $$, expensive; GPS, global positioning system; SAQ, self-administered questionnaire*.

**Table 6:** Domain: Internal factors.

**NCS measurement category/domain**	**Subdomain**	**Data collection method**	**Recommended measure**	**Time/burden/cost**	**Status of recommended measure**	**Notes**	**Visit Type**
Physical health/biologic	Epigenetics	Biospecimens	Venous blood; cervical cells, placenta, buccal oral cells, urine	$$	Needs development		PHS
	Internal effect, HPA disruption, immune function, epigenetics	Biospecimens	Blood, dried blood spots (DBS)	$$	Needs development	DBS needs development	PHS
	Internal effect, HPA disruption	Biospecimens	Saliva, hair	$$	Ready		PHS
	Internal effect, other hormones (sex steroids, DHEA)	Biospecimens	Hair	$$	Needs development	High throughput needs development	PHS
	Internal effect, epigenetics	Biospecimens	Placenta	$$	Ready		PHS
	Oxidative stress	Biospecimens	Urine	$$	Ready		PHS
	Internal effect, mithochondriomics	Biospecimens	Venous blood	$$	Needs development	High throughput needs development	PHS
	Internal effect, exosomes	Biospecimens	Breast milk	$$	Needs development		PHS

*The column Visit Type maps the assessment to data collections that in some cases are mapped to other domains such as Physical Health Systems (PHS) and Social–Emotional–Behavioral (SEB) (see respective chapters in this edition)*.

*$$, expensive*.

This narrative discusses the following:

1) *What do these measurements represent?* This narrative encompasses three different views: discussion of the state of the art for exposure measurements, the importance of an emphasis on biomarkers for exposure assessment as these are the most direct estimate exposure and our view toward obtaining maximum amount of information from specific biospecimens and exposure techniques. For example, a biospecimen that can be collected once and then analyzed for multiple exposures hold more value than a sample, which can only be used to assess one exposure. It has been challenging for most cohorts to determine what measures, when and where, and how. This is especially true across life stage where specimens may be limited, and participant burden is an important consideration.
2) *How can the narrative and grid be used?* The narrative and the grid are divided into domains (listed above), measurement categories, and subdomains. Across all of these, the group assessed data collection method, recommended measures, time, burden and cost, status of recommended measure, and life course assessment timing. Data collection methodologies include questionnaires, biospecimen collection, environmental samples, observations, and extant data usage. Each data collection methodology is linked with a recommended measure, including a specific biospecimen or questionnaire. In order to allow the grid to be relevant to future studies, the status of the recommended measure was also assessed. This allowed for inclusion of methodologies that are not ready for implication yet, but show significant promise for future work. All of these measures are discussed in the context of developmental timing for optimal assessment. The grid was designed for comprehensive longitudinal cohort studies over 21 years. To use this moving forward, there is a context and purpose that could be applied to pull out subsets of the assessments to answer specific questions and or hypothesis. Also, as one reviews cohort studies, such measures and their consistency could be used to facilitate the cross study interpretation and translation.
3) *What types of measurements and tools are employed in the narrative and grid?*
  *Survey measures*. Survey measures such as those listed below are the least expensive construct type, although they do not always provide quantitative exposures and can contribute significantly to participant time burden. They may be administered by study staff or self-administered by the respondent. They may be administered by direct questioning, or privately via computer. Technological advances also allow these to be completed via web or smartphone.
  *Questionnaires*. Questionnaires ask the respondent questions about a given topic. The questions may address themselves, their family or friends, their child, or their home, school, or neighborhood/community. Examples of exposure topics for which questionnaires are recommended include stress, family relationship quality, use of pesticides and consumer products, occupational exposures, sources and perception of noise, and home, school, and neighborhood/community characteristics.
  *Diaries*. Diaries ask the respondent to answer the same questions over time. These are usually left with the respondent to complete at specified time points. Examples of exposure topics for which diaries are recommended include diet and time-place activities.
  *Observational measures*. Observations can be made by a data collector or the participant. In either case, these survey measures cost more than questionnaires or diaries. Observations done by a data collector add staff costs, while observations made by a participant may require educating the participant and may be biased. The types of environmental exposure information that can be captured by observations include indoor and outdoor environmental and safety features of the home, child care/school, or workplace. It may also be difficult to obtain permission to conduct observations of child care/school and work settings.
  *Sample collection with laboratory analysis*. Environmental samples are collected to determine levels of chemical or environmental toxicants in various media (e.g., dust, water, air, soil, food). Sample collection is relatively inexpensive where samples can be left in storage for future case-control study analyses. However, some sample types cannot be stored and must be analyzed immediately. While lab analysis can be costly, some analytical methods can measure many chemicals (see *Multiple Chemical Assessments* discussion below). There is also the issue that just because a chemical is in the environment does not mean it gets into a child's body and impacts health. Sample collection is generally only recommended where there is no other way to assess the exposure, e.g., via survey methods, extant data, or biospecimen collection. As such, the types of exposures for which sample collection is recommended include indoor air particulate matter and air oxidants, and semi-volatile chemicals via vacuum bag dust and dust wipe sample collection.
  *Direct monitoring*. Direct monitoring approaches typically involve devices, which measure an exposure directly and integrate the exposure over time. Examples include measurement of air oxidants via sensors, measurement of noise levels, and physical activity accelerometers. These types of devices can be moderately to very expensive upfront, but do not require subsequent laboratory analysis, and can be left in a home, other environment, or with a participant to determine exposures over specified time periods. Sensor and computer application technologies are evolving rapidly, and smartphone compatible sensors may soon be available.
  *Biomarkers*. Where available, biomarker measures are generally considered more direct exposure measures than survey, ambient monitoring, and sampling approaches. Options for biomarker measures are discussed in more detail below.
  *Geospatial analysis*. Geospatial analyses link addresses to extant data, e.g., EPA air monitoring data or local crime statistics, to estimate exposures to individuals in various locations, e.g., the home/neighborhood, school, or work. Geospatial modeling has been utilized to characterize a number of environmental factors including local food environments (56–58), built environment for physical activity (59), ambient pollution (60), water contamination, and spatio-temporal patterns in crime (61). There has also been a call for the need to include disability-specific items in measures of the built environment (62). While this type of data requires no input from or burden to the participant, costs for developing and maintaining these databases and analyses can be substantial.
  *Exposure models*. Chemical exposures can be modeled from available information, including extant data, findings from survey measures and observations, sampling data from a subset of other participants, etc.
  In the subsequent sections of this report, we describe the domains used in the grid in detail. Chemical and Biological Exposures, Physical Environment, Social Stress, Social Determinants of Health and Effect Modifiers are all discussed below with special care to address subdomains and recommended measures included in the grid.

## Grid Introduction

The Health Measurement Network (HMN) Environmental Domain Working Group developed recommendations for assessment of environmental exposures in the National Children's Study (NCS). The grid summarizes the group's recommendations of measurement for the following five categories: (1) Chemical and biological exposures including organic chemicals (pesticides, organochlorine pesticides, organophosphate pesticides, and metabolites, carbamate pesticides, fungicides, herbicides, brominated flame retardants, disinfection byproducts, environmental tobacco smoke markers, environmental phenols, parabens, perfluorinated compounds, BPA and phthalates, polychlorinated biphenyls, polycyclic aromatic hydrocarbon and metabolites, and volatile organic hydrocarbons), inorganic chemicals (metals, and perchlorate and other anions), and biological exposures (allergens and mold, and microbes); (2) physical environment exposures including community design, physical safety, access to food resources, radiation exposure, ultraviolet radiation, radon, and noise exposure; (3) stress including social support and coping; (4) social determinants of health including socioeconomic status, family relationship quality, parent/caregiver mental health, parenting difficulties, attachment, child care/school characteristics, community violence, social capital, school environment, peers and neighborhood, and mentoring; and (5) modifying factors including diet, obesity, and physical activity. The recommended measures are given from pre-conception through young adulthood. For each subdomain within a category, the grid specifies the recommended data collection method, recommended measure, time/burden/cost, prioritization of source of information, status of the recommended measure, notes about the recommendation as well as the visit type and time of the measurement.

## Importance of the Grid and How It Could Be Used

Based on the discussions above, we developed a grid for assessment of both chemical and non-chemical exposomes (see Tables 1–6).

1. Examples of specific measures, how and when they would be used in a life course cohort study.
2. Identifies state of the art methods and identifies where further development is needed.
3. Consolidates measures for chemical, physical, and social determinants of health in one framework across “environment.”
4. Inclusion of time, burden, and cost also provides insight for alternate measures.
5. Provides a common platform for integrating exposure measures across multiple cohorts and studies.

The grid can also be used to prioritize measures of exposure. Factors to consider in selecting environmental measurements for a specific cohort study include the research question to be answered, budget, population, and exposure scope. Prioritization criteria include value of information obtained from a particular environmental measure, direct relationship of measurement to receptor (primary vs. secondary markers), and ability to provide co-exposure solutions, i.e., the same measurement could provide information on multiple chemicals and exposure routes. For example, integrated measures that show exposure of a chemical over time might have higher prioritization because of the ability to assess the temporality of exposure, especially during pregnancy when more direct measures of exposure profiles may be less feasible. There are many measures that are “ready,” i.e., the methodology is developed, and the recommended measure is available for immediate use. Other prioritization factors include the validation, stability, specificity, and sensitivity of the assays to be used. Although not a key focus of this assessment, the limit of detection is a key element that needs to be matched with the research question. Ease of collection and participant burden is a factor in all prioritization schemes and can vary dramatically between study constructions. This grid and these prioritization criteria can be used to help identify the optimal assessment methods for children's environmental health cohorts.

## Data Availability Statement

The original contributions presented in the study are included in the article/supplementary material, further inquiries can be directed to the corresponding author/s.

## Author Contributions

SV: manuscript facilitator and editor, wrote some sections, and managed tables. MD: NIH investigator leading effort and reviewed all. EC: wrote section on behavioral instruments. TM: wrote sections on tooth analysis. EF: technical support and reviewer. SB: wrote section on built environment. MS: researched references for psychosocial assessment methods. RW: team lead, wrote introductory sections and figures. All authors: contributed to the article and approved the submitted version.

## Conflict of Interest

The authors declare that the research was conducted in the absence of any commercial or financial relationships that could be construed as a potential conflict of interest.
